# Enhancing Nuclear Medicine Practice in Saudi Arabia: Advocating for Comprehensive Guidelines and Local Diagnostic Reference Levels

**DOI:** 10.7759/cureus.54230

**Published:** 2024-02-15

**Authors:** Sarah K Albahiti, Mawya Khafaji, Nadiya Batawil, Norwin Catipay, Khalid Alsafi

**Affiliations:** 1 Radiology, King Abdulaziz University Hospital, Jeddah, SAU; 2 Radiology, Faculty of Medicine, King Abdulaziz University, Jeddah, SAU; 3 Radiology, King Abdulaziz University, Jeddah, SAU

**Keywords:** icrp, radiation saftey, diagnostic reference levels, drl, nuclear medicine

## Abstract

Introduction: Diagnostic reference levels (DRLs) were initially introduced by the International Commission on Radiation Protection (ICRP). It refers to the measured quantity of administered activity (MBq) in nuclear medicine imaging studies and is a type of investigation level. DRL is recommended to prevent excessive radiation exposure to patients while maintaining adequate image quality. It should not be implemented as a dose constraint or dose limit. The Saudi Food and Drug Authority (SFDA) is the primary government body responsible for reporting national diagnostic reference levels (NDRLs) for diagnostic medical imaging technologies in Saudi Arabia. Only NDRLs for computed tomography, general X-ray, and mammography have been published and enforced locally. This study aims to establish local DRLs for nuclear medicine imaging procedures at King Abdulaziz University Hospital, Saudi Arabia, preparing for compliance proof once required by local authorities.

Method: Data were collected from all machines, and six common protocols were studied, with data from 50 patients of standard body size for each identified protocol. The study was conducted retrospectively, and the 50th percentile was then calculated for each scan.

Results: Both protocols for renal scans administered the lowest doses to patients (130 MBq and 148 MBq), respectively. The highest dose administered to patients was found to be in bone scans (1110 MBq).

Conclusion: The study establishes local DRLs for nuclear medicine imaging in our institution. Median activity dosages in renal, thyroid, and parathyroid imaging were comparable to locally and internationally published DRLs. However, they are higher in cardiac and bone imaging compared to local Saudi DRL and DRL in the European Union and the USA, likely due to the adopted protocols. These highlight the need for modifying the protocols to fulfill optimization efforts. These findings serve as a foundation for compliance with future regulatory requirements, ensuring patient safety and maintaining imaging quality in Saudi healthcare.

## Introduction

In Saudi Arabia, 51 nuclear medicine centers operate under the Ministry of Health, governmental sectors, and private hospitals [1]. These centers conduct approximately 37,655 general nuclear medicine investigations and 12,387 cardiac scans annually. According to a 2018 survey, the country is equipped with 21 positron emission tomography/computed tomography (PET/CT) machines, 55 single-photon emission computed tomography/computed tomography (SPECT/CT) machines, and 35 SPECT and gamma cameras [1].

The concept of diagnostic reference levels (DRLs) was initially introduced in publication 73 by the International Commission on Radiation Protection (ICRP) [2] to refer to a readily measurable quantity, typically the administered activity (MBq) in nuclear medicine imaging studies, representing a type of investigation level. Since DRLs are recommended to prevent excessive radiation exposure in patients while maintaining adequate image quality, they should not be utilized as dose constraints or limits [3].

In 1999, the European Commission published Radiation Protection 109. It stated that member states should establish DRLs, considering distinct national or regional circumstances, such as the availability of equipment and training, in accordance with Radiation Protection 180 [4,5]. Following ICRP 73, the ICRP published practical supporting guidelines in 2001 (A1) and ICRP 105 in 2007, both of which supported the establishment of DRLs [6,7]. The most recent publication, ICRP 135, details the existing guidelines and various aspects of DRL establishment, including considerations for conducting national surveys, clinical applications of DRLs, and updating intervals for DRLs [8]. In many countries, DRLs have been established by these instructions [9-18].

While efforts to establish DRLs start with the basic diagnostic imaging protocols that are commonly used in daily practice, more advanced and complex modalities are also needed for DRLs for practice optimization. PET/CT is a hybrid, noninvasive diagnostic procedure used in nuclear medicine and biomedical research [19]. PET imaging utilizes positron-emitting radionuclides with short half-lives concentrated in specific tissues. The annihilation of positrons causes the emission of two 511 keV photons in collinear but opposite orientations, and the detection of these photons permits the molecular imaging of human organs [19,20]. In addition to diagnostic CT images, CT provides data for correcting PET/CT images to account for attenuation caused by photon annihilation in human tissues. By combining CT and PET, valuable information can be obtained regarding disease diagnosis, staging, and follow-up. Because CT and PET utilize ionizing radiation and deliver highly effective doses to patients, optimizing PET/CT radiation dose levels is necessary [16].

Alkhybari et al. [16] published a systematic review of DRL methods for PET/CT and SPECT/CT, identifying significant regional and national discrepancies in the methods used to calculate and report DRLs. The authors recommended more robust standards to improve comparability with international reference levels [16]. DRLs for PET/CT and SPECT/CT were determined by collecting radiation doses from the administered activity (A), measured in MBq, the CT dose in volume CT dose index measured in mGy, and the dose length product measured in mGy multiplied by cm (mGy.cm) [20]. Two distinct measures, the 75th percentile and guidance level, were used to report the DRL values for (A). The 75th percentile method is based on an assessment of the distribution of the median A from participant centers in a national or regional DRL survey. It is used to report the A- and CT-DRLs [4,21]. For standard-sized patients, national guidance levels were determined based on evidence from professional expert organizations. The recommended A dose is reported using guidance levels but not the CT dose [21]. The achievable dose provides an additional standard for optimizing diagnostic imaging without compromising image quality [22,23].

The achievable dose corresponds to the 50th percentile of the national NDRL and is used to determine the dose typically administered in clinical practice. Centers with a local DRL below the 75th percentile should optimize the acquisition protocol and apparatus to approach the achievable dose limit [22,23]. In total, DRLs for 11 protocols frequently used in PET examinations and 22 protocols used in SPECT have been published for clinical benchmarking [24].

Over 5,000 nuclear medicine procedures are conducted annually in Kuwait, and the amount of delivered radiopharmaceutical activity varies up to 20 times among various nuclear medicine departments [14]. In 2021, this variation prompted Alnaaimi et al. to establish NDRLs [14].

In 2022, Fayad et al. [25] conducted a survey involving three facilities, which collectively housed five machines, to establish NDRL for Qatar, focusing exclusively on adults. The NDRL for Qatar was determined to be 20% lower than that for Kuwait in thyroid uptake, 49% in renogram, 50% in renal scintigraphy, 50% in lung, and 57% in parathyroid.

Efforts to establish DRLs for all medical imaging procedures in Saudi Arabia are relatively recent, necessitating the optimization of practices to achieve higher patient safety standards [26]. Al-Qahtani et al. [27] surveyed 10 nuclear medicine departments in different Saudi Arabian hospitals in 2021 and suggested NDRLs for SPECT/CT scans using administration activity (AA) in MBq and SPECT/CT protocols. They compared their proposed NDRL with those of Croatia, the United Kingdom, Korea, Australia, Brazil, and Japan for AA in MBq and also with those of Japan, Nordic countries, the UK, Kuwait, and Switzerland for the CT components of hybrid imaging SPECT/CT devices.

The Saudi Food and Drug Authority (SFDA) is the primary governmental body responsible for reporting NDRL for diagnostic medical imaging technologies in Saudi Arabia, facilitated by the formation of a National Radiation Safety Committee assigned to this task [28].

To date, only the NDRL for CT, general radiography, and mammography have been published and enforced locally [28]. Efforts are underway to establish NDRLs for other modalities, including nuclear medicine, which is highly needed, especially considering that diagnostic imaging is a newly regulated area of medicine in Saudi Arabia.

Although the SFDA has established NDRLs for adult diagnostic CT examinations, NDRLs for SPECT/CT are yet to be established. Consequently, this study aims to establish local DRLs for nuclear medicine imaging procedures at our institution, ensuring readiness for compliance verification once mandated by local authorities.

## Materials and methods

The data were collected from all machines at the Nuclear Medicine Unit of the Department of Radiology at King Abdulaziz University Hospital, Saudi Arabia. The details of the machines are presented in Table 1. Six commonly used protocols were studied, as presented in Table 2. The data comprised information from 50 patients with a standard body size for each identified protocol collected retrospectively.

**Table 1:** Nuclear medicine equipment information.

Manufacturer	Model	Acquired Year	Type
Siemens	Symbia T	2008	Dual Head
General Electric	Discovery N/M/CT670	2014	Dual Head
Philips	Bright View	2010	Dual Head

**Table 2:** Protocols included in the study.

Protocol Name	Radiopharmaceutical	Imaging Agent	Activity (MBq)	Acquisition Time (min)
Bone scan	Technetium 99	Methylene Diphosphonate (MDP)	1110	45
Thyroid scan	Technetium 99	Technetium Pertechnetate (TcO4)	185	20
Parathyroid scan	Technetium 99	Methoxy-IsobutyI-Isonitrile (MIBI)	740	90
Renal MAG	Technetium 99	Mercapto-Acetyltri-Glycone (MAG-3)	185	45
Renal DMSA	Technetium 99	Dinercaptosuccinic Acid (DMSA)	185	20
Myocardial perfusion scan (rest/stress)	Technetium 99	Methoxy-IsobutyI-Isonitrile (MIBI)	Rest 407	Rest 16
			Stress 1036	Stress 16

Data were collected manually using a data sheet developed to extract patient details from the logbooks. One technician was chosen to fill in the information and a second one to verify the inputted data. The six most repeated protocols in the unit were identified as the most commonly utilized and included in this analysis, in accordance with ICRP [1].

Patient inclusion criteria were based on average-sized patients that already attending the unit and received the scan. The study did not involve additional exposure of patients and relied on patient records. The collected data did not include patient identification for anonymization and privacy.

Patient demographic data included sex, age, weight, and radiation dose quantity in terms of AA (MBq). This survey specifically focused on the activity of radioactive pharmaceuticals administered, excluding the dose associated with CT exposure.

Statistical analyses

The minimum (min), maximum (max), median, first quartile (Q1), third quartile (Q3), and standard deviation (SD) values were calculated as descriptive statistics. All statistical analyses were performed using Statistical Product and Service Solutions (SPSS, version 21; IBM SPSS Statistics for Windows, Armonk, NY).

## Results

Data from 300 patients were included in this retrospective study. Forty percent of the patients were male, whereas 60% were female, with an average weight of 64.4 ± 28.2 kg. The dose distributions (minimum, maximum, mean, SD, first quartile (Q1), second quartile (Q2), and third quartile (Q3)) of the six protocols are presented in Table 3.

**Table 3:** Local DRLs for six nuclear medicine procedures performed.

Injected Activity MBq		Min	Max	Mean	SD	25th	50^th^ (Median)	75th	DRL
Bone scan		962	1184	1107.04	41.5	1073	1110	1110	1110
Myocardial perfusion scan	Rest	407	444	412.92	13.7	407	407	407	407
	Stress	925	1036	963.48	35.8	925	962	962	962
Parathyroid scan		666	814	721.50	54.0	666	703	777	703
Renal scan (MAG-3)		55.5	185	125.80	55.3	74	130	185	130
Renal scan(DMSA)		74	185	133.20	46.7	74	148	185	148
Thyroid scan		148	222	172.42	31.4	148	148	194	148

Comparisons of the DRLs from this study with those of other countries for protocols where the associated DRLs exist are shown in Table 4.

**Table 4:** Comparison of our local DRLs to that of other countries: Qatar, Kuwait, Japan, Australia, the UK, the USA, and the EU. UK: United Kingdom, USA: United States of America, EU: European Union [5,29,30].

Procedure	Radiopharmaceutical	The present study	10 centres in Saudi	Qatar	Kuwait	Japan	Australia	UK	USA	EU
Bone scan	Tc-99m	1110	815.4	740	944	944.4	920	800	848-1185	499.5-1110
Myocardial perfusion scan	Tc-99m	407-962	1017.5	926	976	900	620	800	945-1402	300-1480
										300-1500
Parathyroid scan	Tc-99m MIBI	703	869.5	384	900	800	900	900	----	399.6-899.1
Renal scan	Tc-99m (MAG-3) dynamic	130	365.4	189	370	377.8	500	300	283-379	99.9-370
Renal scan	Tc-99m (DMSA) static	148	185	101	200	207.4	200	80	189-289	69.9-181.3
Thyroid scan	Tc-99m	148	194.3	195	185	237	215	80	----	72-222

## Discussion

In medical imaging, there exists a notable variation in patient doses among service providers, with nuclear medicine procedures showing a potential eightfold variation depending on factors such as protocol, machine, and staff experience [27].

This variation made it essential to establish DRLs in every institution and continuously audit the local or national DRLs for the sake of patient safety.

The present study showed that the median activity administered in renal imaging (technetium 99m MAG3 and technetium 99m DMSA) was 130 and 148 MBq, respectively (Table 3). This was lower than the published reference data (Table 4), as 50% of the renal patients in this study were in the pediatric age group. Weight-based dosing guidance is used in our center to calculate pediatric AA [31].

The median dose activity in the current study for one-day myocardial perfusion imaging was 1369 MBq, which is comparable to data from some European countries (France, Italy, and Luxembourg) [5]. This value is higher than the locally published Saudi data (1017.5 MBq), where the doses administered for one- and two-day protocols are combined.

Eighty-six percent of the bone scans in the current study were performed on patients with breast and prostate cancers, utilizing the whole body SPECT examination protocol (1110 MBq) to evaluate remote skeletal or extra-axial lesions that might be overlooked in local or targeted SPECT/CT [32-34]. This variation in the bone scan protocol may explain the higher median value in the current study compared to local Saudi and Gulf region data. However, these data were comparable to that of the USA and EU (Table 4).

The variation in the doses between practices and countries will always exist since these doses represent the practice, not individual patient doses. DRLs should contribute to enhancing safety nuclear medicine guidelines without hindering daily workflow or staff satisfaction. Acceptable variation in DRLs exists, provided that it does not compromise patient safety or image quality. It has been reported as high as eight times, depending on the protocol, machine, and staff experience [27].

These findings highlight the necessity for optimizing protocols, particularly the bone scan protocol, initially set at 1110 MBq compared to the benchmark. Therefore, the protocol was adjusted, and dose reduction was achieved. The waiting period for the scan ranged from two to four hours, using the whole body SPECT/CT protocol for all oncology patients. Consequently, the adjustments were implemented, reducing the injected dosage to 925 MBq and shortening the waiting time from four to two hours for the protocol.

This study underscores the need for optimizing myocardial perfusion scan protocols. Therefore, a two-day protocol was adapted to reduce the cumulative dose from 1332 MBq to an adjusted 888 MBq over two days (with 444 MBq for rest and stress phases).

## Conclusions

In summary, the current study demonstrates comparable median activity dosages in renal, thyroid, and parathyroid imaging to both local and internationally published DRLs. However, the higher cardiac and bone imaging doses, akin to DRLs in some EU countries and the USA, are likely influenced by adopted protocols, suggesting a need for modification to optimize radiation exposure.

The current study had several limitations, including a small dataset, single-center, and the absence of PET services. Additionally, the reported dose was only for the activity, and the effective radiation dose for the CT component in hybrid examinations was not calculated. The future work will address these limitations as phase 2 of the DRL development. In addition, it is important to establish pediatric DRLs for the common protocols used for that population. Despite these limitations, findings from this study underscore the importance of ongoing efforts to refine protocols and align with evolving safety standards in nuclear medicine.

## References

[1] Alqarni A, Farghaly H, Nasr H (2019). Current situation of nuclear medicine in Saudi Arabia. Int J Sci Res.

[2] International Commission on Radiological Protection (1996). Radiological Protection and Safety in Medicine. ICRP Publication.

[3] Cho SG, Kim J, Song HC (2017). Radiation safety in nuclear medicine procedures. Nucl Med Mol Imaging.

[4] (2024). European Commission. Radiation Protection 109: guidance on diagnostic reference levels for medical exposures. https://energy.ec.europa.eu/system/files/2014-11/109_en_1.pdf.

[5] European Commission (2024). Radiation Protection N° 180: Diagnostic Reference Levels in Thirty-Six European Countries. http://energy.ec.europa.eu/system/files/2015-01/RP180%2520part2_0.pdf.

[6] International Commission on Radiological Protection (2007). Radiation Protection in Medicine. ICRP Publication.

[7] (2001). Diagnostic reference levels in medical imaging: review and additional advice. Ann ICRP.

[8] Vañó E, Miller DL, Martin CJ (2017). ICRP Publication 135: diagnostic reference levels in medical imaging. Ann ICRP.

[9] Hart D, Wall BF (2005). UK nuclear medicine survey 2003-2004. Nucl Med Commun.

[10] Gray L, Torreggiani W, O'Reilly G (2008). Paediatric diagnostic reference levels in nuclear medicine imaging in Ireland. Br J Radiol.

[11] Roch P, Aubert B (2013). French diagnostic reference levels in diagnostic radiology, computed tomography and nuclear medicine: 2004-2008 review. Radiat Prot Dosimetry.

[12] Abe K, Hosono M, Igarashi T (2020). The 2020 national diagnostic reference levels for nuclear medicine in Japan. Ann Nucl Med.

[13] Song HC, Na MH, Kim J, Cho SG, Park JK, Kang KW (2019). Diagnostic reference levels for adult nuclear medicine imaging established from the National Survey in Korea. Nucl Med Mol Imaging.

[14] Alnaaimi MA, Alduaij MA, Shenawy FA (2022). National diagnostic reference levels for nuclear medicine in Kuwait. J Nucl Med Technol.

[15] Willegaignon J, Braga LF, Sapienza MT (2016). Diagnostic reference level: an important tool for reducing radiation doses in adult and pediatric nuclear medicine procedures in Brazil. Nucl Med Commun.

[16] Alkhybari EM, McEntee MF, Brennan PC, Willowson KP, Kench PL (2019). Diagnostic reference levels for (18) F-FDG whole body PET/CT procedures: results from a survey of 12 centres in Australia and New Zealand. J Med Imaging Radiat Oncol.

[17] Wachabauer D, Beyer T, Ditto M (2022). Diagnostic reference levels for nuclear medicine imaging in Austria: a nationwide survey of used dose levels for adult patients. Z Med Phys.

[18] Becker MD, Butler PF, Siam M (2019). U.S. PET/CT and gamma camera diagnostic reference levels and achievable administered activities for noncardiac nuclear medicine studies. Radiology.

[19] Jallow N, Christian P, Sunderland J, Graham M, Hoffman JM, Nye JA (2016). Diagnostic reference levels of CT radiation dose in whole-body PET/CT. J Nucl Med.

[20] Kwon HW, Kim JP, Lee HJ (2016). Radiation dose from whole-body F-18 fluorodeoxyglucose positron emission tomography/computed tomography: nationwide survey in Korea. J Korean Med Sci.

[21] International Commission on Radiological Protection (2007). The 2007 Recommendations of the International Commission on Radiological Protection. https://www.icrp.org/publication.asp?id=ICRP%20Publication%20103.

[22] Iball GR, Bebbington NA, Burniston M (2017). A national survey of computed tomography doses in hybrid PET-CT and SPECT-CT examinations in the UK. Nucl Med Commun.

[23] Alessio AM, Farrell MB, Fahey FH (2015). Role of reference levels in nuclear medicine: a report of the SNMMI dose optimization task force. J Nucl Med.

[24] Lima TV, Gnesin S, Ryckx N, Strobel K, Stritt N, Linder R (2018). Swiss survey on hybrid imaging CTs doses in nuclear medicine and proposed national dose reference levels. Z Med Phys.

[25] Fayad H, Ahmed S, Khatib AE, Ghujeh A, Aly A, Kharita MH, Al-Naemi H (2023). National diagnostic reference levels for nuclear medicine in Qatar. J Nucl Med Technol.

[26] Aloufi KM, Alhazmi FH, Abdulaal OM, Qurashi AA (2020). Towards the establishment of diagnostic reference levels in Saudi Arabia: review and opinion. Egypt J Radiol Nucl Med.

[27] Al-Qahtani SM, Alidasroos MA, Alkhybari EM (2023). The establishment of national diagnostic reference levels for adult SPECT-CT in Saudi Arabia. J Radiol Prot.

[28] (2024). National diagnostic reference levels. https://www.sfda.gov.sa/sites/default/files/2023-02/NDRL-En.pdf.

[29] (2024). Notes for guidance on the clinical administration of radiopharmaceuticals and use of sealed radioactive sources. GOV.UK.

[30] National Council on Radiation Protection and Measurements. Scientific Committee 4-3 on Diagnostic Reference Levels and Achievable Doses, and Reference Levels in Medical Imaging: Recommendations for Applications in the United States (2012). Reference Levels and Achievable Doses in Medical and Dental Imaging: Recommendations for the United States. https://ncrponline.org/publications/reports/ncrp-report-172/.

[31] Poli GL, Torres L, Coca M, Veselinovic M, Lassmann M, Delis H, Fahey F (2020). Paediatric nuclear medicine practice: an international survey by the IAEA. Eur J Nucl Med Mol Imaging.

[32] Tulik M, Tulik P, Kowalska T (2020). On the optimization of bone SPECT/CT in terms of image quality and radiation dose. J Appl Clin Med Phys.

[33] Abikhzer G, Gourevich K, Kagna O, Israel O, Frenkel A, Keidar Z (2016). Whole-body bone SPECT in breast cancer patients: the future bone scan protocol?. Nucl Med Commun.

[34] Rager O, Nkoulou R, Exquis N (2017). Whole-body SPECT/CT versus planar bone scan with targeted SPECT/CT for metastatic workup. Biomed Res Int.

